# Adding an orange to the banana bag: vitamin C deficiency is common in alcohol use disorders

**DOI:** 10.1186/s13054-019-2435-4

**Published:** 2019-05-10

**Authors:** Paul E. Marik, Amanda Liggett

**Affiliations:** 1 Division of Pulmonary and Critical Care Medicine, Eastern Virginia Medical School, 721 Fairfax Ave, Suite 423, Norfolk, VA 23507 USA; 2 Department of Medicine, Eastern Virginia Medical School, 721 Fairfax ave, Norfolk, VA 23507 USA

**Keywords:** Alcohol use disorder, Thiamine, Vitamin C, Delirium tremens, Alcohol withdrawal

## Abstract

**Background:**

At least a third of the world’s population consumes alcohol regularly. Patients with alcohol use disorders (AUDs) are frequently hospitalized for both alcohol-related and unrelated medical conditions. It is well recognized that patients with an AUD are thiamine deficient with thiamine replacement therapy being considered the standard of care. However, the incidence of vitamin C deficiency in this patient population has been poorly defined.

**Methods:**

In this retrospective, observational study, we recorded the admission vitamin C level in patients with an AUD admitted to our medical intensive care unit (MICU) over a 1-year period. In addition, we recorded relevant clinical and laboratory data including the day 2 and day 3 vitamin C level following empiric treatment with vitamin C. Septic patients were excluded from this study.

**Results:**

Sixty-nine patients met the inclusion criteria for this study. The patients’ mean age was 53 ± 14 years; 52 patients (75%) were males. Severe alcohol withdrawal syndrome was the commonest admitting diagnosis (46%). Eighteen patients (26%) had cirrhosis as the admitting diagnosis with 18 (13%) patients admitted due to alcohol/drug intoxication. Forty-six patients (67%) had evidence of acute alcoholic hepatitis. The mean admission vitamin C level was 17.0 ± 18.1 μmol/l (normal 40–60 μmol/l). Sixty-one (88%) patients had a level less than 40 μmol/l (subnormal) while 52 patients (75%) had hypovitaminosis C (level < 23 μmol/l). None of the variables recorded predicted the vitamin C level. Various vitamin C replacement dosing strategies were used. A 1.5-g loading dose, followed by 500-mg PO q 6, was effective in restoring blood levels to normal by day 2.

**Conclusion:**

Our results suggest that hypovitaminosis C is exceedingly common in patients with an AUD admitted to an intensive care unit and that all such patients should receive supplementation with vitamin C in addition to thiamine. Additional studies are required to confirm the findings of our observational study and to determine the optimal vitamin C dosing strategy.

## Background

In 2016, 32% of the world’s population reported the regular consumption of alcohol [1]. Generally, the amount of alcohol consumed is greater by males and those in high socioeconomic countries. Between 2006 and 2010, the annual number of alcohol-associated deaths in the USA was approximately 88,000 or 9.8% of all US deaths [2]. Applying Diagnostic and Statistical Manual of Mental Disorders 5th edition (DSM-5) [3] diagnostic criteria, in 2012–13 36·0% of male and 22·7% of female adults in the USA met the criteria for alcohol use disorders (AUD) at some time in their lives [4]. Patients with AUD are commonly hospitalized for both alcohol-related and unrelated medical disorders [5]. These patients usually have a poor balanced diet and exhibit deficiencies in essential vitamins and nutrients during acute illness. Traditionally, patients in the USA hospitalized with an AUD have been treated with a “banana bag” containing 100 mg thiamine as well as multivitamins. In their paper entitled “Unpeeling the Evidence for the Banana Bag” which reviews the management of alcohol-associated vitamin and electrolyte deficiencies, Flannery et al. suggest abandoning the banana bag and instead providing routine supplementation of high-dose thiamine (200–500 mg IV thiamine every 8 h), IV magnesium sulfate, and oral folic acid [6]. These authors do not recommend routine supplementation with vitamin C. However, few studies have evaluated vitamin C levels in patients with AUDs. It is well recognized that thiamine deficiency is common in patients with AUD [6]. Like thiamine, vitamin C is a water-soluble vitamin with limited body stores [7]. Furthermore, it is exceedingly uncommon for patients to be deficient in a single nutrient, with most malnourished patients being deficient in multiple vitamins and nutrients. Considering that most patients with AUDs have a diet poor in fresh fruits, one would anticipate that vitamin C deficiency would be common in these patients. Furthermore, low serum levels of vitamin C are common in critically ill patients [7, 8]; this likely compounds the vitamin C nutritional deficiency of alcoholic patients admitted to the ICU. Since vitamin C is an important anti-oxidant, is an essential cofactor for numerous biological reactions, and is required for normal cognitive function, it is likely that a deficiency of this vitamin would have important clinical consequences [7, 9]. While a number of case reports of vitamin C deficiency in patients with AUD have been reported [10–14], few studies have specifically evaluated vitamin C levels in patients with AUDs. In 1981, Majumdar et al. measured leukocyte vitamin C levels in 25 chronic alcoholics [15]. In this study 24 (96%), patients were found to be vitamin C deficient. In 1987, Baines reported on the incidence of vitamin B and C deficiency in a series of 35 patients with alcohol-related illnesses [16]. In this study, 31% of patients were thiamine deficient while 91% were deficient in vitamin C. Bergheim et al. assessed the nutritional status of 76 “middle-class alcohol consumers” hospitalized with different stages of alcoholic liver disease [17]. These authors reported that between 55 to 64% of patients with alcohol-related liver disease had decreased plasma vitamin C concentrations. More recently, Lux-Battistelli evaluated the incidence of vitamin C deficiency in 47 patients undergoing alcohol detoxification [18]. In this study, only 30% of patients had normal vitamin C levels. As part of our sepsis protocol in which we routinely administer adjuvant vitamin C [19], we noted that AUD patients were frequently vitamin C deficient. Based on this observation, we now routinely measure vitamin C levels in our AUD patients and administer vitamin C together with thiamine, magnesium, and folate in the dosages as suggested by Flannery et al. [6]. In this paper, we report the incidence and risk factors for hypovitaminosis C in a cohort of AUD patients admitted to our medical ICU.

## Methods

This study was an electronic health record (EHR)-based retrospective clinical study. The Eastern Virginia Medical School (EVMS) Institutional Review Board approved the study protocol (19-01-Feb0003). This study was conducted at Sentara Norfolk General Hospital, a tertiary care referral hospital affiliated with EVMS and the only tertiary care facility in the Hampton Roads area serving a population of approximately 1.8 million people. Starting in January 2018, all patients admitted to the EVMS Critical Care Medicine service in the Medical ICU (MICU) at Sentara Norfolk General Hospital with an alcohol-related primary diagnosis and/or a history of chronic alcohol abuse had a vitamin C level measured on admission. The total ascorbic acid level was performed by high-pressure liquid chromatography (HPLC) with electrochemical detection by LabCorp (Burlington, NC, USA). The specimens were collected in a serum separation gel tube, protected from light and transported on ice. The specimens were frozen prior transport to the local reference laboratory (LabCorp, Norfolk, VA, USA). Patients received high-dose thiamine and magnesium as per the protocol of Flannery et al. [6]. In addition, patients received vitamin C supplementation which was dosed at the discretion of the treating team, with attempts to repeat the vitamin C levels on day 2 and day 3.

The inclusion criteria for this study included patients admitted to our MICU with an AUD in whom an admission vitamin C level was measured. An AUD was defined according to the DSM-5 criteria, which required at least two of 11 symptoms to be present for the diagnosis of an AUD [2, 3]. AUD patients who were septic on admission were treated with the hydrocortisone, ascorbic acid thiamine (HAT) protocol and were excluded from this study [16] as were patients less than 18 years or over 90 years of age. We queried our EHR (EPIC, Verona, WI, USA) from January 2018 to December 2018 to identify patients who met the inclusion criteria for this study. The patients’ clinical and demographic data including the age, sex, admitting diagnosis, requirement for mechanical ventilation, and baseline laboratory tests including s-creatinine, BUN, magnesium, hemoglobin, mean cell volume (MCV), platelet count, INR, lactate, and liver function tests were recorded. As per our ICU protocol, vitamin C levels were measured at baseline and repeated after 24 and 48 h. Severe alcohol withdrawal syndrome (SAW) was defined as delirium tremens, withdrawal seizure, or clinically diagnosed with severe withdrawal [20]. Acute alcoholic hepatitis was defined as an admission aspartate aminotransferase (AST) greater than the upper limit of normal of our laboratory (AST > 37 IU/l). Dexmedetomidine and benzodiazepines were administered according to our hospital’s CIWA protocol (Clinical Institute Withdrawal Assessment for Alcohol Scale) [2, 4, 21, 22]. Survival to hospital discharge was recorded.

### Data analysis

The patients’ deidentified clinical and laboratory data were recorded in an electronic spreadsheet. Summary statistics were used to describe the clinical data and presented as mean ± SD or percentages as appropriate. Chi-squared analysis with Fisher’s exact test (when appropriate) and Student’s *t* test (Mann-Whiney *U* test for non-normal distributions) was used to compare data between groups, with statistical significance declared for probability values of 0.05 or less. Pearson correlation was performed to determine the relationship between two continuous variables. Statistical analysis was performed using NCSS 11 (Kaysville, Utah).

## Results

Sixty-nine patients met the inclusion criteria for this study. The patients’ mean age was 53 ± 14 years; 52 patients (75%) were males. The most frequent diagnosis associated with AUD was severe alcohol withdrawal syndrome (46%), followed by complications associated with alcoholic cirrhosis—encephalopathy or gastrointestinal bleeding (26%) and acute alcohol intoxication associated with substance abuse (13%). Nine (13%) patients required mechanical ventilation. Forty-six patients (67%) had evidence of acute alcoholic hepatitis. Eighteen patients had an elevated serum lactate concentration on admission (> 2.2 mmol/l). Forty patients (58%) received an infusion of dexmedetomidine while 44 (64%) received bolus lorazepam according to our CIWA protocol. The hospital mortality was 6%; all patients who died had a preexisting diagnosis of cirrhosis.

The mean admission vitamin C blood level was 17.0 ± 18.1 μmol/l. The mean level was 18.3 ± 18.9 μmol/l in the male patients and 12.6 ± 15.1 μmol/l in the female patients (NS). Sixty-one (88%) patients had a level less than 40 μmol/l (subnormal), and 52 patients (75%) had a level less than 23 μmol/l (hypovitaminosis C) with 29 patients (42%) having a level less than 11 μmol/l (severe vitamin C deficiency) [8, 23]. Eighteen patients (26%) had an undetectable vitamin C level on admission. Clinical, demographic, and laboratory data of the entire cohort as well as those with a vitamin C level < 23 μmol/l and > 23 μmol/l are presented in Table 1. None of the variables recorded differentiated between patients with a vitamin C level < 23 μmol/l (hypovitaminosis C) and those with a level > 23 μmol/l. The mean vitamin C level was 19.0 ± 20.2 μmol/l in the patients with severe alcohol withdrawal syndrome, 14.0 ± 20.7 in the patients with alcoholic cirrhosis, 13.6 ± 10.8 in the patients with drug/alcohol intoxication, and 8.9 ± 9.3 in the patients with AUD and chronic obstructive pulmonary disease/congestive heart failure (no difference between groups). Twenty-two patients (32%) had an admission magnesium level < 1.8 mg/dl (normal value 1.8–2.5 mg/dl). There was a poor correlation between the admission vitamin C level with the magnesium level (*r* = 0.26), albumin (*r* = 0.001), and BMI (*r* = − 0.1). Various vitamin C replacement dosing strategies were used based on the preference of the treating physician and the ability of the patients to take oral medications. Follow-up (i.e., days 2 and 3) vitamin C levels were available on 47 patients (68%). The follow-up levels stratified by the dosing regimen are provided in Table 2.

**Table 1:** Primary diagnosis and clinical and laboratory data grouped according to vitamin C level on admission

	All *n* = 69	Vitamin C < 23 μmol/l *n* = 52	> 23 μmol/l* n = 17
Age (years)	53 ± 14	54 ± 13	50 ± 15
AWS (*n*, %)	32 (46%)	23 (44%)	9 (53%)
Cirrhosis (*n*, %)	18 (26%)	13 (25%)	5 (29%)
Drug + AI (*n*, %)	9 (13%)	8 (15%)	1 (6%)
COPD/CHF (*n*, %)	5 (7%)	5 (10%)	1 (6%)
BMI (kg/m2)	25.2 ± 5.2	25.2 ± 5.4	25.2 ± 6.0
BUN (mg/dl)	14.6 ± 9.7	14.5 ± 9.9	14.9 ± 9.3
Magnesium (mg/dl)	1.8 ± 0.4	1.9 ± 0.4	1.6 ± 0.2
AST (IU/l)	84 ± 84	80 ± 87	98 ± 73
Albumin (g/dl)	3.3 ± 0.8	3.3 ± 0.8	3.4 ± 0.7
MCV (fl)	89 ± 14	89 ± 15	91 ± 11
Lactate (mmol/l)	2.9 ± 3.1	2.9 ± 3.0	2.9 ± 3.3

*AI* alcohol intoxication, *AWS* alcohol withdrawal syndrome, *AST* aspartate aminotransferase, *BMI* body mass index, *BUN* blood urea nitrogen, *COPD* chronic obstructive pulmonary disease, *CHF* congestive heart failure, *MCV* mean cell volume

*No significant difference between groups

**Table 2:** Baseline and follow-up vitamin C levels (μmol/l) according to treatment strategy

	Baseline	Day 2	Day 3
500 mg PO q 6 h (*n* = 14)	22.4 ± 23.6	57.0 ± 26.2	91.2 ± 36.2
1.5 G IV then 500 PO q 6 h (*n* = 19)	11.7 ± 13.9	119.4 ± 43.3	99.4 ± 31.8
1.5 IV daily (*n* = 8)	9.8 ± 9.5	144.2 ± 54.3	169.7 ± 68.2
1.5 IV q 6 h (*n* = 6)	23.3 ± 21.9	294.4 ± 82.8	317.8 ± 111.4

## Discussion

In this small observational study, we have demonstrated that the vast majority of patients with an AUD are vitamin C deficient with 42% being severely deficient (vitamin C level < 11 μmol/l). Our findings are in agreement with previous studies [15–18] and are not unexpected considering the poor diet of most alcoholics. Fruits such as oranges, lemons, grapes, strawberries, papaya, kiwi, cantaloupe, grapefruit, and mango are good sources of vitamin C and likely to be consumed in small quantities if at all by alcoholics. However, it should be noted that Carr et al. reported subnormal levels of vitamin C in a cohort of non-septic patients admitted to the ICU (mean level of 20.8 ± 8.9 μmol/l) [8]. It is therefore difficult to distinguish between subnormal vitamin C levels due to dietary deficiency and that due to critical illness and metabolic consumption [7]. However, vitamin C deficiency is common in non-hospitalized alcoholic patients [18], suggesting that dietary deficiency may play an important role in the subnormal serum levels of vitamin C in non-septic hospitalized alcoholic patients [15, 16]. Nevertheless, regardless of the cause, our study indicates that patients with an AUD admitted to the ICU are at a high risk of hypovitaminosis C and should receive replacement therapy.

Deprivation of vitamin C in the human diet leads to scurvy which can be a life-threatening disorder. However, critically ill patients can also develop vitamin C deficiency due to increased metabolic consumption, glomerular hyperfiltration, decreased gastrointestinal absorption, and decreased recycling of dehydroascorbate to ascorbic acid [7, 8]. Overt physical signs of scurvy may not develop until very low plasma vitamin C concentrations have been reached, perhaps as low as 3–5 μ/mol/l. [24] However, the WHO recognizes an early or “latent” form of scurvy, in which symptoms are non-specific and include fatigue, malaise, weakness, leg pain, muscle ache, and mild cognitive dysfunction [23, 25, 26]. In the alcoholic patient, these non-specific symptoms may be attributed to alcohol withdrawal, liver dysfunction, or other nutritional disorders. As vitamin C deficiency progresses, collagen synthesis becomes impaired and connective tissue becomes weakened due to lack of hydroxyproline causing easy bruising, petechial hemorrhages, bleeding in the gums, bone weakening and pain, fracture, and poor wound healing [12, 24, 27].

In the 1950s, a series of classic experiments performed by Raymond Adams and Maurice Victor unequivocally demonstrated that thiamine was required to reverse the ophthalmoplegia associated with Wernicke’s syndrome [28, 29]. Following these seminal studies, thiamine has been used both therapeutically and prophylactically to prevent Wernicke’s syndrome in patients with AUD. In their detailed observational studies, it was noted that the confusional state associated with Wernicke’s syndrome frequently did not abate until the patients took a full diet which included fruit and fruit juices (the Kemper Rice Diet) [29]. Adams postulated that other nutritional deficits may have played a role in Wernicke’s syndrome; this was likely vitamin C. Most clinicians are unaware of the vital role of vitamin C in neuro-cognitive function [30].

Vitamin C is concentrated almost 100-fold in neurons with the highest concentration found in the hippocampus and the frontal cortex [31, 32]. Vitamin C is a pivotal antioxidant in the brain and plays an important role in neuromodulation [32–34]. Vitamin C affects synaptic neurotransmission by preventing neurotransmitters binding to receptors, by modulating their release and reuptake, and also acting as a cofactor in neurotransmitter synthesis [35]. Vitamin C plays a role in presynaptic re-uptake of glutamate and inhibits binding of this neurotransmitter to the NMDA receptor [36–38]. Vitamin C is postulated to play an important role in cognitive function. In a systematic review, Travica et al. reported higher mean vitamin C concentrations in cognitively intact participants as compared to participants who were cognitively impaired [39]. Low levels of vitamin C have been implicated in the development of Alzheimer’s disease and other neurodegenerative diseases [33–35, 40]. In these neurodegenerative diseases, a clear link has been established between vitamin C deficiency and oxidative-induced neuronal death [41]. The altered neuropsychiatric function has been reported in both the acute and chronic forms of scurvy [23, 27, 42]. In a population-based study, Pearson et al. demonstrated that patients with low serum vitamin C levels had an increased incidence of mild cognitive impairment [43]. In this study, the odds of mild cognitive impairment were twice as high for those with a serum vitamin C level below 23 mol/L. Voigt et al. demonstrated that vitamin C levels were significantly lower in the CSF of patients with septic encephalopathy as compared to control patients, with the vitamin C levels being correlated with the severity of neurologic symptoms [44]. These data suggest that low vitamin C levels may play a role in the pathophysiology of the confusional state and cognitive impairment of patients with Wernicke’s encephalopathy and those with alcohol withdrawal syndromes.

There appears to be a bi-directional relationship between ethanol and vitamin C. Ethanol is known to have direct toxic effect on the enterocytes of the gastrointestinal tract and thus may interfere with the intestinal absorption of various nutrients including vitamin C. Furthermore, alcohol has been demonstrated to significantly increase the urinary loss of vitamin C, likely contributing to the vitamin C deficiency noted in alcoholics [45]. Animal studies have demonstrated that both short- and long-term pretreatment with vitamin C before alcohol dosing significantly lowered blood alcohol levels [46, 47]. Chen et al. studied the effects of short- and long-term vitamin C supplements on plasma alcohol clearance in clinically healthy male subjects [48]. In this study, both short- and long-term pretreatment with vitamin C significantly enhanced the clearance of plasma alcohol. Similarly, Susick et al. reported that vitamin C increased ethanol clearance and improved tests of motor coordination and intellectual function after acute alcohol ingestion in healthy volunteers [49]. These studies suggest that vitamin C may play a role in alcohol detoxification. The mechanism by which vitamin C enhances ethanol metabolism is not quite clear. It has been suggested that vitamin C, by functioning as an electron donor, spares the NAD/NADH system and thus accelerates the conversion of alcohol to its metabolites [50]. Acetaldehyde, the first metabolite of ethanol metabolism, has been implicated in ethanol-induced hepatotoxicity [51, 52]. Furthermore, the induction of hepatic cytochrome P4502E1 by ethanol increases the oxidative stress in hepatocytes [53].

Animal studies suggest that vitamin C may limit alcohol-induced oxidative injury and thereby protect hepatocytes against the toxic effects of alcohol [53–57]. The potential for vitamin C to limit ethanol-induced hepatotoxicity has not been investigated in humans; however, this simple and cheap intervention holds much promise and prospective studies are required to address this issue.

The optimal vitamin C dosing strategy in patients with an AUD is unclear. It is uncertain if patients should be dosed to achieve normal (40–60 μmol/l) or supranormal levels. In patients with sepsis, supra-normal levels (trough levels of approximately 200–300 μmol/l) are required to modulate the inflammatory response [7, 9, 19, 58]. As vitamin C may have a beneficial effect in patients with acute alcoholic hepatitis [53–57], it is possible that supranormal levels are required in these patients. At a minimum, dosing should achieve normal vitamin C levels as quickly as possible.

## Conclusions

In this retrospective, observational study which included patients with an AUD who were admitted to an ICU for both alcohol- and non-alcohol-related medical conditions, 75% had hypovitaminosis C. Previous research has demonstrated that vitamin C increases ethanol clearance and may reduce alcohol-induced hepatotoxicity. These data suggest that critically ill patients admitted to the ICU with chronic alcoholism should be treated with vitamin C. Additional studies are required to confirm the findings of this small observational study. Furthermore, the optimal dosing strategy and both the short- and long-term benefits of vitamin C supplementation in this patient population remain to be determined.
